# Evaluating the Ethical Beliefs of the Peruvian Consumer After the COVID-19 Pandemic: An Approach from the Perspective of Green Consumption Orientation and Green Purchasing Intention

**DOI:** 10.3390/bs16071126

**Published:** 2026-07-06

**Authors:** Miluska Villar-Guevara, Dany Yudet Millones-Liza

**Affiliations:** 1 Escuela Profesional de Administración, Facultad de Ciencias Empresariales, Universidad Peruana Unión, Salida a Arequipa Km. 6 Chullunquiani, Av. Héroes de la Guerra del Pacífico, Juliaca 21100, Peru; 2 Escuela Profesional de Administración, Facultad de Ciencias Empresariales, Universidad Peruana Unión, Km 19.5 de la Carretera Central, Ñaña, Lurigancho-Chosica, Lima 15102, Peru; dannie@upeu.edu.pe

**Keywords:** consumer ethical beliefs, green consumption orientation, green purchasing intention, consumer psychology, sustainable consumption, green consumers, environmentally conscious consumers

## Abstract

Consumer behavior evolves unexpectedly, and understanding new perspectives according to market trends is a key factor in identifying the most commonly recurring consumption demands. In this context, this study aimed to examine the associations among green consumption orientation, consumer ethical beliefs and green purchasing intention. Based on a quantitative and explanatory study with the participation of 411 Peruvian consumers, this study demonstrated that green consumption orientation has a direct and negative association with consumers’ ethical beliefs, while green consumption orientation has a direct and positive association with green purchasing intention. Finally, green purchasing intention has a direct and negative association with the ethical beliefs of Peruvian consumers. These findings reveal a reconfiguration of the ethical framework of Peruvian consumers, where green consumption orientation acts as a catalyst for the intention to purchase eco-friendly products, but at the same time generates tensions with traditional ethical beliefs. This phenomenon suggests that consumers are developing new value systems that prioritize environmental sustainability over conventional ethical considerations, which represents both a challenge and an opportunity for companies seeking to position themselves in the green market.

## 1. Introduction

Consumer behavior is constantly evolving. Initially, studies helped to establish the reasons why consumer behavior changed, identifying factors such as personal preferences, price, quality, and payment method (Palomino et al., 2020; Perčić et al., 2023). However, this behavioral trend changed after the arrival of the COVID-19 health crisis, as consumers moved toward purchasing products that, beyond meeting their needs, promote environmental sustainability through actions that reduce food waste, with a focus on quality, well-being, and fairness, resulting in conscious consumption (Ahmad et al., 2023; Valenzuela-Fernández et al., 2022). As a result, current consumer behavior patterns are focused on an interest in organic products, better purchase planning, and dietary changes, that is, greater consumption of healthy foods (Chilón-Troncos et al., 2024a; García-Salirrosas et al., 2025) and less of ultra-processed products (Silva-Paz et al., 2024; Vásquez et al., 2022). The changes mentioned are a response to consumer trends, such as ethical consumerism, the influence of social and environmental concerns, the impact of behavioral consistency, and personal and cultural factors (Boccia et al., 2019; Hennigs et al., 2017).

Ethical beliefs play an important role, as they are influenced by internal factors encompassing personal beliefs, and external factors such as transparency regarding the company’s ethical investments and sustainable practices; thus, previous research refers to ethical beliefs as a purchasing decision factor, which acts more strongly on the younger population where ethics predominate as an added value of a brand and as a driver of business innovation (Chiou & Pan, 2008; E. Lopes et al., 2020). While consumer ethics is a powerful predictor of purchase intention, this prediction can vary depending on psychological and situational barriers (Chatterjee et al., 2022); therefore, ethical beliefs become a strategic asset that strengthens consumer preference, loyalty (García-Salirrosas et al., 2024), and purchase intention, with the latter being extensively analyzed in the scientific community due to the need to understand market opportunities. In this context, studies agree that an orientation toward green consumption is important for gaining a deeper understanding of the attitudes and intentions behind ecological consumption, which is influenced by environmental concerns and value orientation (Ayoun & Schmitz, 2024; J. M. Lopes et al., 2024).

Specifically in the Peruvian context, before the pandemic, technological and consumer trends represented 8.1% of sales, a figure that rose after the arrival of the pandemic to over 61% (Ortiz-Chávez et al., 2024). In this scenario, consumer preference for ecological, organic, and eco-friendly products predominates; this translates into a greater sense of responsibility, from product selection to product management—that is, reducing waste and ensuring the sustainability of packaging (Armas-Castañeda et al., 2025; Cristóbal et al., 2022; García-Salirrosas et al., 2022). Thus, it is stated that in the pandemic scenario, the Peruvian consumer felt the need to acquire ecological products in order to protect their health. As they became more informed, they maintained a positive trend toward increased awareness and more environmentally responsible consumption. Evidence of this is reflected in a report that indicates that 76% of the companies operating in Peru noted increased interest among Peruvians for ecological products (Armas-Castañeda et al., 2025); this explains why, beyond the environmental benefits of eco-friendly consumption, Peruvians sought to satisfy their need for uniqueness and improve their self-image (Afshar & Jia, 2018).

While there appears to be growing consumer interest in organic products, there are shortcomings in aligning what the market offers with consumer expectations (Chilón-Troncos et al., 2024b). One possible explanation is that a large percentage of consumers lack knowledge about the real benefits and characteristics of organic products, which affects their purchasing decisions (J. M. Lopes et al., 2024). Moreover, in some cases, despite the fact that environmentally conscious consumers are willing to pay more for an eco-friendly product, in reality, this purchase does not occur due to the cost barrier and doubts about the true effects of the product to be consumed. This is due to both a lack of credibility in eco-labels and to the limited availability of these products, which also interrupts the frequency of purchase (Nicolae, 2024; Terlau & Hirsch, 2015). In this sense, there is an urgent need to continue exploring consumer behavior in terms of promoting health and as a contribution from society toward achieving the Sustainable Development Goals, specifically those associated with life on land and responsible production and consumption.

In general terms, the pandemic acted as a catalyst for consumer behavior, and various studies have analyzed consumer consumption and purchasing trends. However, in the Peruvian context, a knowledge gap has been identified regarding the mechanisms that help understand how ethical beliefs have been redefined following the pandemic and how consumer knowledge can be translated into eco-friendly purchasing intentions. To address this gap, this study aimed to examine the associations among green consumption orientation, consumer ethical beliefs and green purchasing intention. The results obtained will provide evidence of consumer behavior based on ethical beliefs, which will be useful for managing investment decisions, product development, and strategies. Similarly, this study will support the Peruvian government in designing specific policies that promote sustainable consumption, thus giving greater impetus to society’s participation in the transition toward responsible consumption patterns.

## 2. Literature Review and Hypothesis Development

### 2.1. Green Consumption Orientation and Consumer Ethical Beliefs

Green consumption orientation (GCO) refers to consumers’ willingness to incorporate sustainability, environmental responsibility, and social responsibility (Cruz et al., 2026) criteria into their purchasing decisions (Yaqub et al., 2023). It is not simply about choosing “green” products because they are fashionable or trendy, but about understanding consumption as an act with direct consequences for the health of the planet and future generations (Jain et al., 2024). Essentially, this approach seeks to balance satisfying consumer needs, business profitability, and protecting the planet, promoting a more ethical and ecological lifestyle (Ogiemwonyi & Jan, 2023). This is behavior reflected in everyday actions such as preferring recyclable products, reducing the use of disposable plastics, opting for renewable energy sources, or supporting companies that demonstrate responsible waste management (Yaqub et al., 2023).

Previous studies have demonstrated the existence of various factors that are associated with the trend toward eco-friendly consumption, with environmental awareness being one of the most decisive factors capable of affecting consumer behavior and purchase intentions regarding environmentally friendly purchases (Liang et al., 2024; Wierzbiński et al., 2021). However, as eco-friendly consumption becomes more normalized and consumers adopt it as a social or environmental obligation, its association with ethical beliefs may vary (Ogiemwonyi & Jan, 2023). Similarly, recent studies indicate that consumers with greater knowledge of green consumption tend to prioritize products and brands that demonstrate a genuine commitment to sustainability and environmental protection over their ethical beliefs when making purchasing decisions (Mancuso et al., 2024; Tan et al., 2022; Tsvakirai, 2024). This apparent contrast may be explained by variations in actual consumer behavior, whereby purchasing decisions are shaped by individuals’ orientation toward green consumption, which, in turn, may be associated with their ethical beliefs. Consequently, these beliefs may vary depending on the consumption context; for example, consumers may place greater priority on product enjoyment than on environmental sustainability (Herédia-Colaço & Coelho Do Vale, 2018; Klink-Lehmann et al., 2023).

Furthermore, consumers can experience green confusion, as the practice of greenwashing has now been exposed. This trend can affect or diminish consumer perception; it only takes one company to tarnish its environmental image for consumers to become skeptical and lose trust, leading to negative emotions that can alter their beliefs (J. M. Lopes et al., 2023; Xiao et al., 2021). Based on the above, this study establishes the following hypothesis:

**H1.**  *Green consumption orientation is negatively associated with consumer ethical beliefs.*

### 2.2. Green Consumption Orientation and Green Purchasing Intention

Green purchasing intention (GPI) can be understood as the probability that a person will decide to acquire a product or service in the near future. In this sense, the greater the intention, the more likely it is that the purchase will take place (Caceres-Polloyqueri et al., 2026; Shabnam et al., 2021). In addition, Berki-Kiss and Menrad (2022) suggest that when it comes to organic products, this intention reflects the consumer’s willingness to choose sustainable options over traditional ones; thus, the literature maintains that intentions represent a key factor in anticipating human behavior, taking into account that people usually make rational decisions based on the information they have (Duong et al., 2023; Larios-Gómez et al., 2021; Nekmahmud et al., 2022).

On the other hand, when analyzing the link between green consumption orientation and eco-friendly purchase intention, the literature argues that green consumption orientation acts as a key driver in shaping eco-friendly purchase intention; this means that consumers who maintain a high level of concern for the environment and sustainability tend to opt for eco-friendly products, even when they are more expensive or difficult to find on the market (Nascimento & Maria Correia Loureiro, 2024; Srisathan et al., 2024). To understand the dynamics of the variables mentioned, previous research indicates that a focus on green consumption generates greater environmental awareness and, consequently, greater environmental concern. These factors positively affect eco-friendly purchasing intentions, as consumers have a high tendency to adopt eco-friendly purchasing habits (Hoang & Hoang, 2023; Joshi, 2016). Thus, the following hypothesis is proposed:

**H2.**  *Green consumption orientation is positively associated with green purchasing intention.*

### 2.3. Green Purchasing Intention and Consumer Ethical Beliefs

Consumer ethical beliefs (CEBs) are described as a system of principles, values, and judgments that guide how individuals evaluate what is right or wrong in their personal and social behavior (Lozano et al., 2026); these form an essential part of the decision-making process and actual action (Khan & Abbas, 2023). Within the Theory of Planned Behavior, beliefs are antecedents of intentions and, ultimately, of behavior rather than consequences of intention; therefore, intentions become a more immediate determinant of behavior, and intentions are shaped by behavioral, normative, and control beliefs (Ajzen & Schmidt, 2020). However, ethical beliefs do not fall into any of these categories, which results in the absence of an explicit mechanism for understanding how an intention might reshape this construct. By revisiting cognitive dissonance theory (Festinger, 1957), this study argues that the association works in the opposite direction under certain conditions: when a consumer’s intention to make an eco-friendly purchase is limited by barriers such as price or accessibility (Laheri, 2020; Liu et al., 2024), and the resulting inconsistency between intention and actual behavior generates psychological distress. To resolve this dissonance, consumers do not necessarily abandon the purchase intention itself; rather, they reconfigure or weaken the ethical beliefs that conflict with their limited behavior, a rationalization mechanism rather than a direct cognitive–behavioral pathway (Eckhardt et al., 2010; Zane et al., 2016).

It is expected that all eco-friendly purchases will transcend individual decisions and become collective values; a positive cultural shift toward a conscious and ecological lifestyle is also anticipated (Talwar et al., 2021). However, this disconnect between intention and behavior is reinforced by difficulties in accessing information, which generates a gap between ethical intentions and responsible purchasing behavior, since it is known that consumers have difficulty translating their ethical intentions into concrete actions due to a lack of prior knowledge (Azzopardi & Van Der Sluis, 2024). Based on the above, this study proposes the following hypothesis:

**H3.**  *Green purchasing intention is negatively associated with consumer ethical beliefs.*

**Figure 1:**
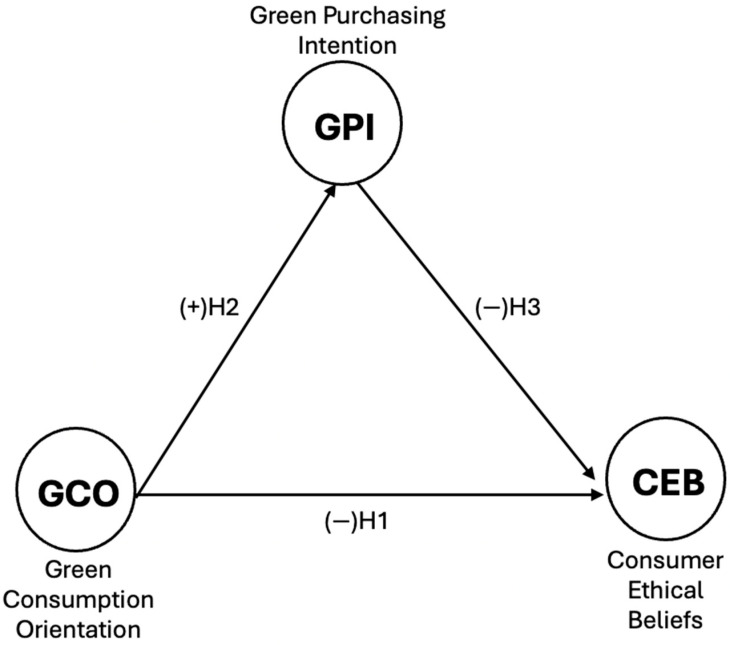
Proposed hypothetical model that reflects the links between consumer ethical beliefs (CEB), green consumption orientation (GCO), and green purchasing intention (GPI).

Taking into account the hypotheses mentioned above, the conceptual model resulting from the study can be visualized in the graphic representation of Figure 1.

## 3. Materials and Methods

### 3.1. Study Design

This study aimed to examine the association between green consumption orientation, consumer ethical beliefs and green purchasing intention. To achieve the purpose of the study, an explanatory design based on the Structural Equation Model (SEM) was used (Ato et al., 2013).

### 3.2. Population and Sample

Organic products are known for promoting beneficial habits for both the consumer and the environment. This type of consumer is more attentive to product characteristics that will lengthen their life. While COVID-19 certainly marked a turning point in perspectives on reusable items and services, it also made us more cautious about the services we offer and the things we buy. Therefore, these consumers are reevaluating their ethical values, prioritizing products and services that reflect greater social and environmental responsibility.

A total of 645 Peruvian consumers were invited to participate in the study; however, only 411 complete and valid surveys were collected for coding. Specifically, this study focused on participants who met certain inclusion criteria, such as (1) being Peruvian; (2) being at least 18 years old; (3) being an occasional or frequent consumer of organic products (these potential participants were recruited from popular ecological communities in Peru, whose members shared this characteristic); and (4) being a dependent worker (providing services within an employment relationship with an employer and receiving remuneration in accordance with current labor legislation) or independent worker (offering services independently, without an employment relationship, managing their own professional activity and tax obligations). This last criterion ensured that the sample maintained active participation in the workforce and, therefore, possessed the necessary experience to make informed judgments about the variables analyzed. Consequently, this condition was used exclusively to define the target population and not as a sociodemographic variable in the study. Those who did not meet these characteristics were excluded from participation. Incomplete responses, inattentive response patterns, and questionnaires with excessive missing data or inconsistencies in responses were excluded to ensure data quality and methodological rigor (a total of 234 surveys were excluded).

This entire process was carried out using non-probability convenience sampling (Otzen & Manterola, 2017). Table 1 details the characteristics of the collected sample, which corresponds to consumers with ages ranging from 18 to 75 years (M = 26.6; SD = 8.1). The sample was balanced between men and women (49.4% and 50.6%, respectively); however, the majority (74.5%) of participants were from Gen Z (18–28 years), and most consumers declared themselves as being of the Adventist (43.6%) and Catholic (29.7%) faith.

### 3.3. Ethical Considerations

To ensure the rigor and reliability of this study, it was conducted exclusively with adults (over 18 years of age). It is important to note that all participants were informed about the research objectives and how their information would be used. Before completing the survey, they provided their informed consent. The questionnaire was self-administered and anonymous to increase data accuracy. The survey was conducted via Google Forms and distributed through social media platforms such as WhatsApp, Facebook, and Instagram (the online questionnaire was available for approximately two months, from 28 July 2024). The research was conducted after review and approval of the study by the Ethics Committee of the Graduate School of a private Peruvian university (Approval Code: 2024-CEEPG-00134; 11 June 2024).

### 3.4. Measuring Instruments, Process of Back-Translation and Cultural Adaptation

This study followed a structured three-stage process to ensure cultural validity during the adaptation phase of the original instruments. In the first stage, two independent bilingual experts (English–Spanish) performed a forward and back translation to verify conceptual equivalence (Brislin, 1986; Harkness et al., 2010). In the second stage, a panel of five experts in marketing and environmental sustainability evaluated the content validity of the adapted items, confirming their representativeness for the Peruvian context. In the third stage, a pilot test was conducted with a subsample of 30 consumers to evaluate the comprehensibility of the items and the consistency of the responses, allowing for minor adjustments to the wording.

The questionnaire was structured in three sections. The first section presented the questionnaire instructions and the informed consent form, which required participants to declare “I agree to participate”. The second section collected the respondents’ personal data (age, sex, and religious affiliation). Finally, the third section contained the three measurement scales based on the respondent’s opinion or belief. All measurement scales were of a one-dimensional structure. CEB coding was used for 13 items (example item: “Purchasing a mobile phone from an illegal vendor”) of Consumer Ethical Beliefs (Dai et al., 2011); for this metric, 5-point Likert-type response options were used (1 = I firmly believe it is wrong; 2 = I believe it is wrong, but there could be exceptions; 3 = Undecided; 4 = I believe it is right, but there could be exceptions; 5 = I firmly believe it is not wrong). GCO coding was used for 5 items (example item: “I book only hotels with a sustainability certification”) of Green Consumption Orientation (Yaqub et al., 2023), and GPI coding was used for 3 items (example item: “I intend to purchase environmentally friendly products because of its environmental concern”) of Green Purchasing Intention (Sharma et al., 2020). For GCO and GPI, the same 5-point Likert-type response options were used (1 = Strongly disagree; and 5 = Strongly agree). All constructs achieved a high level of reliability, with Cronbach’s alpha values of 0.931, 0.819, and 0.901, respectively.

### 3.5. Statistical Analysis

Partial Least Squares–Structural Equation Modeling (PLS–SEM) was used to carry out hypothesis testing, thus taking advantage of PLS–SEM as a comprehensive approach that allows multivariate statistical analysis to be carried out in order to obtain information regarding the measurement and structural components for examining the relationships among the variables (Hair et al., 2010). In addition, PLS–SEM was used in this study because it facilitates the construction of theories (Hair et al., 2011). All constructs were operationalized as reflective following the theoretical rationale that their indicators are manifestations of the underlying latent variable; that is, the construct causally produces its indicators, and interchangeability among indicators is assumed (Hair et al., 2019a). The choice of PLS–SEM over Covariance-Based SEM (CB–SEM) is further justified by the exploratory and predictive nature of the study, the non-normality of observed data distributions, and the presence of formative-adjacent constructs in the theoretical framework (Chin, 2010; Hair et al., 2011).

## 4. Results

Partial least squares–structural equation modeling (PLS-SEM) was used to perform the statistical analysis of the data. The PLS-SEM evaluation consisted of two stages: assessment of the psychometric properties of the measurement scale, such as reliability, convergent validity, and discriminant validity; and hypothesis testing using the structural equation model.

### 4.1. Evaluation of the Measurement Model

According to Hair et al. (2017), to evaluate the measurement model, an estimate of construct reliability (composite reliability and Cronbach’s alpha) and validity (discriminant and convergent validity) was proposed. Cronbach’s alpha (α) values ranged from 0.819 to 0.931, and the threshold value of 0.7 fell below these values (Hair et al., 2017). Similarly, composite reliability (CR) values ranged from 0.855 to 0.943, which were above the suggested value of 0.7 (Kline, 2015). Based on these findings, all constructs were considered error-free, and construct reliability was established (see Table 2).

Average Variance Extracted (AVE) and factor loadings must be tested for convergent validity (Hair et al., 2017). According to Table 2, most factor loadings are above the suggested value of 0.7; some were accepted by decimal approximation (CEB8 was accepted because its value was acceptable relative to the other values that were part of the construct), and item GCO2 was excluded because it had a loading much lower than the established threshold. Furthermore, Table 2 shows that AVE scores range from 0.548 to 0.836 and are above the threshold value of 0.5. These results adequately satisfy convergent validity for all constructs.

### 4.2. Discriminant Validity

To determine discriminant validity, the Heterotrait–Monotrait (HTMT) ratio was considered (Hair et al., 2017). The HTMT results are provided in Table 3, which shows that the threshold value of 0.90 is greater than the value for each construct (Henseler et al., 2015). Except for the HTMT value between GCO and GPI, which slightly exceeded the conservative threshold of 0.90 (HTMT = 0.905), it remained below the more permissive criterion frequently reported in contemporary PLS-SEM studies involving conceptually related constructs. Given the theoretical proximity between GCO and GPI, this result suggests substantial conceptual relatedness without indicating construct redundancy. Therefore, discriminant validity is established based on these findings. These results confirm the validity and reliability of the measurement model. Consequently, the evaluation of the structural model can proceed.

### 4.3. Structural Model Analysis

The proposed hypotheses were tested using PLS-SEM. Predictive relevance values were used for model fitting. Redundancy values with cross-validation (R^2^) represent the predictive relevance of the model. R^2^ values must be greater than 0 for model accuracy (Hair et al., 2014; Henseler et al., 2015). R^2^ values were determined using the blindfolding method, where all values of the endogenous construct were greater than 0, indicating model accuracy. Table 4 shows the endogenous latent variables and their respective R^2^ values.

**Figure 2:**
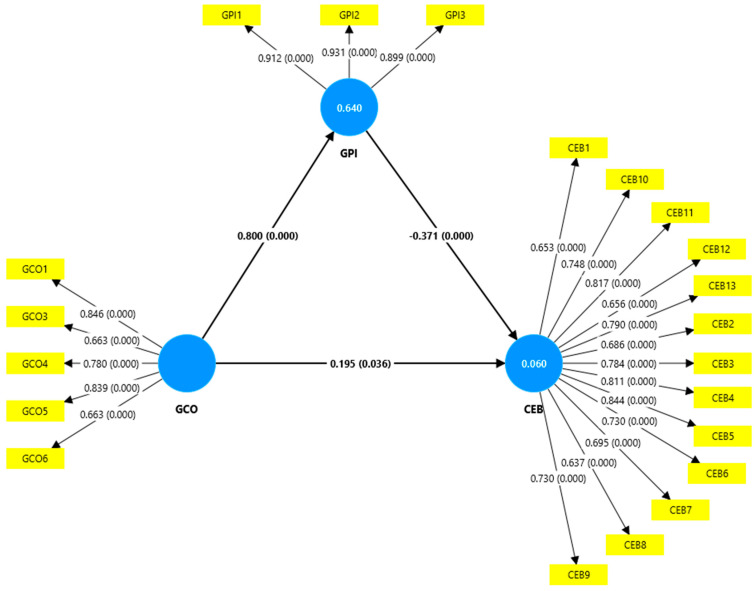
Structural equation modeling that reflects the links between consumer ethical beliefs (CEBs), green consumption orientation (GCO), and green purchasing intention (GPI).

The path coefficient values, *p*-value, and t-statistics were used to accept and reject the hypotheses, as shown in Figure 2 and Table 5. The strength of the relationship between the variables can be examined through the path coefficient values. Path coefficient values close to +1 indicate a strong relationship, and vice versa (Hair et al., 2016). The *p*-values and t-statistics refer to the acceptance and rejection of the proposed hypotheses. In this study, the conceptual model contains three hypotheses. The results of the tested hypotheses are summarized in Table 5.

H1 was accepted, which proposed that green consumption orientation (GCO) has a direct and negative association with the ethical beliefs of the Peruvian consumer (CEB) (β = −0.101; *p* = 0.025; t = 2.249). Hypothesis H2, which proposed that green consumption orientation (GCO) has a direct and positive association with green purchasing intention (GPI) (β = 0.800; *p* < 0.000; t = 29.909), was accepted, as was H3, which proposed that green purchasing intention (GPI) has a direct and negative association with the ethical beliefs of Peruvian consumers (CEB) (β = −0.371; *p* < 0.000; t = 3.613). Thus, all hypotheses were confirmed, and these results indicate that consumers oriented toward green consumption may have a negative view of traditional ethical beliefs, considering that these do not adequately address current problems related to environmental association, the preservation of natural resources, or responsible consumption; therefore, they need to be redefined. Furthermore, it is confirmed that consumers highly oriented toward green consumption show a greater predisposition to buy eco-friendly products. This demonstrates that their values related to environmental stewardship association not only their beliefs but also their purchasing decisions, strengthening their preference for sustainable options in the market. Finally, Peruvian consumers with an intention to purchase environmentally conscious products may perceive that traditional ethical beliefs are not sufficiently relevant to addressing environmental problems; therefore, it would be necessary to update them to adapt to the new demands of more conscious and sustainable consumption.

## 5. Discussion

### 5.1. Discussion of the Results

This study aimed to examine the associations among green consumption orientation, consumer ethical beliefs and green purchasing intention. The results show that green consumption orientation is negatively associated with the ethical beliefs of Peruvian consumers. To support this claim, studies have been identified demonstrating that green consumption orientation currently represents a sign of environmental responsibility (Carbajal-Rubio et al., 2024), thus becoming a consumption obligation that is not necessarily linked to ethical beliefs, but rather more precisely associated with environmental management, ecological services, energy conservation, and the intention to protect the environment (Akhtar et al., 2021; Ghvanidze et al., 2016). The results of this research, which indicate a negative association, appear to contradict this; however, there is evidence explaining that consumers with ethical ideologies prioritize their universal moral principles over particular circumstances (Johnstone & Tan, 2015); another precedent that supports the findings of this research establishes that negative consumer perceptions of what is truly a green or ecological product can often hinder ethical consumption (Zou & Chan, 2019), especially in an environment where consumption has become an obligation and people with an ethical mindset do not usually make ethical purchases (Gacanin & Wagner, 2019; Ogiemwonyi & Jan, 2023; Travassos et al., 2023); in general terms, this translates into the need to achieve a positive attitude toward green consumption among consumers in order to generate an important link between purchasing decisions and ethical considerations.

On the other hand, this study has shown that a green consumption orientation is positively associated with green purchasing intention. This is supported by the fact that, in recent years, an urgent need has been identified for those involved in consumption decisions to be responsible for making more environmentally friendly decisions, adopting a culture of respect translated into the promotion of sustainability that becomes tangible in the intention to purchase eco-friendly products (Moraga et al., 2024; Nguyen et al., 2021; Yadav & Pathak, 2017). Moreover, there are theories that support purchase intention, such as the theory of planned behavior, which explains an individual’s intentions based on an event, belief, or subjectivity. Specifically, the orientation toward green consumption is the consumer’s predisposition to adopt a consumption practice that mitigates environmental impact and promotes sustainability (Albornoz et al., 2024; J. M. Lopes et al., 2024). Another factor supporting the identified findings is that when a consumer maintains a positive orientation toward green consumption, whether as a matter of responsibility or as a result of the associated of ecological role models, their intention to purchase ecological products is greater (De Silva et al., 2025).

Similarly, it has been shown that green purchasing intention is negatively associated with consumer ethical beliefs. The results align with previous research that states that consumers hold diverse perceptions regarding eco-friendly products, so their ethical beliefs may be affected by external factors such as distrust in green marketing, corporate opportunism, or even the true contribution of eco-friendly products to the environment (Harjadi & Gunardi, 2022; Lu et al., 2015). To better understand the negative impact of green buying on ethical beliefs, Okengwu (2023) and Tian et al. (2022) explain that complex interactions exist between a consumer’s intention to make eco-friendly purchases and their ethical judgment. The desire to make eco-friendly purchases can alter ethical beliefs and moral principles, as the conviction of what is right and wrong depends largely on the consumer’s perception of eco-friendly purchasing. Furthermore, the current literature establishes that consumers’ ethical beliefs are influenced by cultural context, business ethics, and other general perceptions (Bartels & Onwezen, 2014; Khan & Abbas, 2023).

### 5.2. Theoretical and Practical Implications

This research contributes to the theoretical growth in the field of consumer behavior, strengthening the link between consumers’ ethical beliefs, their orientation toward green consumption, and their intention to purchase environmentally friendly products. In this sense, this study has made a significant contribution to the development of theories related to these topics, expanding their literary understanding and deepening our knowledge of consumer behavior. Similarly, this research is innovative because it provides scientific support for the proposed and subsequently validated hypotheses, a hypothetical model that had not been previously studied, thus opening up new avenues of research. Furthermore, the research could benefit academics in the field by providing valuable insights into recent literature.

From a practical perspective, the results of this research have significant implications for companies seeking to design optimal strategies based on consumer behavior. Recognizing consumers’ ethical beliefs would allow for a more comprehensive understanding of their potential purchasing orientations. Some of these ethical beliefs might include only consuming/buying from socially responsible companies that uphold a fair labor ethic, reject deceptive practices, respect culture and identity, and maintain environmental awareness. These perceptions of right and wrong are not only associated with purchasing decisions, but also shape how brands are perceived and ultimately associated with their market performance in the Peruvian context. Brands that recognize and value these cultural and ethical principles have the opportunity to develop stronger, more lasting, and more genuine connections with their customers.

On the other hand, this research also proposes that green consumption orientation is positively associated with green purchasing intention, which translates into essential evidence for designing new marketing and sales tactics, particularly for innovative and environmentally conscious companies. Understanding consumers’ perceptions of green consumption orientation allows companies to create more meaningful and authentic purchase and repurchase experiences that align with their expectations, which, in turn, strengthens brand loyalty and affinity. This approach not only generates sustainable competitive advantages, but also contributes to consolidating corporate reputation, as today’s consumers increasingly value consistency between the environmental values that companies promote and the actual practices they implement.

### 5.3. Limitations and Future Research

Despite the relevant findings, this study has certain limitations that must be considered. First, the sample focused exclusively on Peruvian consumers, which restricts the possibility of generalizing the results to other cultural and economic contexts. Furthermore, while the use of structural equation modeling (PLS-SEM) has allowed for the identification of significant relationships between variables, this methodology does not include an in-depth exploration of the qualitative factors that could influence the evolution or redefinition of Peruvian consumers’ ethical beliefs.

Another significant limitation lies in the cross-sectional nature of the collected data, which prevents analysis of how the orientation toward green consumption and the intention to purchase eco-friendly products vary over time. In this regard, in post-pandemic scenarios, it is suggested that future research adopt longitudinal designs to assess changes in consumer attitudes and ethical values. It would also be valuable to explore the effect of green marketing strategies and public policies on promoting responsible consumption in Peru, which would allow for comparative studies in other countries with similar socioeconomic contexts.

A significant limitation of this study lies in the age distribution of the sample, which was predominantly composed of Generation Z consumers (18–28 years; 74.5%) followed by Millennials (29–44 years; 20.2%), while older age groups were considerably underrepresented. This concentration could limit the generalizability of the findings, given that the literature on sustainable consumption suggests that environmental perceptions, ethical beliefs, and eco-conscious purchasing intentions can vary significantly across generations. Consequently, the results obtained may more closely reflect the characteristics, values, and behavioral patterns of younger consumers who tend to exhibit higher levels of environmental awareness and familiarity with sustainability discourse. Therefore, future research should aim for more balanced samples across generational cohorts and incorporate comparative or invariance analyses to examine intergenerational differences in environmentally responsible consumption behavior more accurately.

A potential methodological limitation of this study relates to the assessment of discriminant validity between the GCO and GPI constructs. Although the HTMT value obtained (0.905) is only slightly above the conservative threshold of 0.90 suggested for conceptually related constructs, this result could reflect the theoretical proximity between the two variables rather than a genuine lack of conceptual discrimination. Nevertheless, future research should strengthen this assessment by incorporating complementary procedures, such as HTMT inference using bootstrap confidence intervals, paying particular attention to verifying that these intervals do not include the value of 1.00, as recommended by Henseler et al. (2015). Furthermore, it would be pertinent to complement the analysis with the Fornell and Larcker (1981) criterion and other convergent and discriminant validation strategies to provide more robust methodological evidence on the empirical differentiation of closely linked constructs within explanatory models of sustainable behavior.

Although this study provides evidence of a significant structural model, the variance explained for ethical beliefs is modest (R^2^ = 0.060). According to Hair et al. (2019b), relatively low values may be acceptable depending on the phenomenon under study and the complexity of human behavior. This means that consumers’ ethical beliefs may be influenced by psychological, cultural, social, and contextual factors that were not included in this study’s model, which represents a limitation attributable to the instrument used, which was designed to capture the core constructs of the extended TPB model and did not include items to measure other mediating or moderating mechanisms, since the theoretical framework did not anticipate the need to test the psychological process underlying the association between green purchase intention and ethical beliefs. In light of the findings, future studies could include scales of perceived cognitive dissonance as a mediating variable, with the aim of testing whether the negative association of green purchase intention on ethical beliefs operates specifically through this rationalization mechanism. Similarly, we propose analyzing price sensitivity and the tendency to rationalize as moderating variables—the former to assess whether this relationship is stronger among consumers with financial constraints, and the latter to identify which individual differences predict a greater propensity to resolve dissonance by weakening ethical beliefs.

Finally, the results of this study open up an important debate about an apparent disconnect between the variables analyzed, highlighting the existence of other factors that are associated with ethical beliefs. Therefore, future research could focus on qualitative studies that explore how consumers with ethical beliefs perceive the authenticity of green consumption. Furthermore, segmented research is proposed, focusing on consumers based on their lifestyle and psychological motivators.

## 6. Conclusions

This study has allowed for a deeper understanding of the associations among green consumption orientation, consumer ethical beliefs and green purchasing intention within the post-COVID-19 pandemic context. The results show that consumers with a strong inclination toward sustainable consumption practices tend to question traditional ethical frameworks, perceiving them as insufficient to address current environmental challenges. This evidence highlights the need to rethink ethical beliefs, integrating principles more aligned with sustainability and responsible consumption. The findings also confirmed an association between a greater orientation toward green consumption and a stronger intention to purchase eco-friendly products. This demonstrates that environmentally conscious consumers are willing to prioritize these types of products, even if they involve a higher economic cost. Furthermore, the study suggests that a high intention to purchase eco-friendly products can foster a critical perspective on conventional ethical beliefs, promoting the need to update ethical frameworks surrounding consumption.

One of the most striking findings is that, in the study population, a greater orientation toward green consumption and a greater purchase intention can be negatively associated with traditional ethical beliefs. This means that as environmental responsibility increases, consumers tend to move away from traditional ethical frameworks. A possible explanation for this lies in the Theory of Basic Human Values presented by Schwartz (2012), who argues that values are organized through a circular motivational continuum, an environment where opposing sectors give way to internal tensions. This same theory refers to universalism, specifically nature conceptualized as the preservation of the environment, being in a motivational position opposed to tradition (values, cultural beliefs, family, compliance with norms). In this context, this study suggests that Peruvian consumers have developed a strong consumption orientation when making purchases, preferring to be guided by the values of universalism and nature. These values, due to their motivational structure, create tension with traditional values. This process does not imply a rejection of ethics itself, but rather a reconfiguration of the ethical value system where environmental responsibility emerges as an alternative catalyst that partially displaces conventional norms. This is highly relevant in the post-pandemic context, where the pandemic spurred this reorientation of values among Peruvian consumers, strengthening concerns about sustainability in relation to traditional ethical orientations.

Finally, these results have important implications for both businesses and society at large. Brands wishing to connect with this consumer segment must strengthen their ecological identity and ethical commitment through consistent and transparent communication strategies. It is recommended for future research to expand the sample to other cultural and economic contexts and to implement longitudinal studies that allow for observing the evolution of ethical beliefs and sustainable purchasing intentions over time.

## Figures and Tables

**Table 1:** Sociodemographic profile of 411 consumers of environmentally friendly products.

Characteristic	Category	Frequency	Percentage (%)
Sex	Male	203	49.4
	Female	208	50.6
Generation/Age range	Gen Z (18–28 years)	306	74.5
	Millennials (29–44 years)	83	20.2
	Gen X (45–60 years)	20	4.9
	Baby Boomers (61–75 years)	2	0.4
Religious inclination	Adventist	179	43.6
	Catholic	122	29.7
	Evangelical	25	6.0
	Another religion	28	6.8
	Non-religious	57	13.9

**Table 2:** Convergent validity results.

Construct	Item	Factor Loading	Cronbach’s Alpha	CR	AVE
Consumer Ethical Beliefs (CEBs)	CEB1	0.653	0.931	0.943	0.548
	CEB2	0.686			
	CEB3	0.784			
	CEB4	0.811			
	CEB5	0.844			
	CEB6	0.730			
	CEB7	0.695			
	CEB8	0.637			
	CEB9	0.730			
	CEB10	0.748			
	CEB11	0.817			
	CEB12	0.656			
	CEB13	0.790			
Green Consumption Orientation (GCO)	GCO1	0.846	0.819	0.855	0.582
	GCO3	0.663			
	GCO4	0.780			
	GCO5	0.839			
	GCO6	0.663			
Green Purchasing Intention (GPI)	GPI1	0.912	0.901	0.902	0.836
	GPI2	0.931			
	GPI3	0.899			

Note: The convergent validity results ensured acceptable values (factor loading, Cronbach’s alpha, and composite reliability (CR) ≥ 0.70 and Average Variance Extracted (AVE) > 0.5).

**Table 3:** Heterotrait–Monotrait relationship (HTMT).

Construct	CEB	GCO	GPI
CEB			
GCO	0.119		
GPI	0.222	0.905	

**Table 4:** R^2^ of the endogenous latent variables.

Construct	R^2^
Green Purchasing Intention (GPI)	0.640
Consumer Ethical Beliefs (CEBs)	0.060

**Table 5:** Results of the structural model.

H	Hypothesis	Original Sample (O)	Sample Mean (M)	Standard Deviation (STDEV)	T Statistics (|O/STDEV|)	*p*-Values	Decision
H1	GCO → CEB	−0.101	−0.108	0.045	2.249	0.025	Supported
H2	GCO → GPI	0.800	0.801	0.027	29.909	0.000	Supported
H3	GPI → CEB	−0.371	−0.383	0.103	3.613	0.000	Supported

## Data Availability

Data can be made available upon written request to the corresponding author of this publication.

## Abbreviations

The following abbreviations are used in this manuscript:

CEBs	Consumer Ethical Beliefs
GCO	Green Consumption Orientation
GPI	Green Purchasing Intention
COVID-19	Coronavirus Disease 2019
M	Median
SD	Standard Deviation
H	Hypothesis
HTMT	Heterotrait–Monotrait
AVE	Average Variance Extracted
CR	Composite Reliability
PLS–SEM	Partial Least Squares–Structural Equation Modeling
